# Cross-species transmission of host adaptation of feline leukemia virus between domestic cats and the wild felid *Leopardus guigna*

**DOI:** 10.1186/s13567-026-01770-6

**Published:** 2026-06-13

**Authors:** Cristobal Castillo-Aliaga, Irene Sacristán, Camila J. Stuardo, Constanza Napolitano, Ezequiel Hidalgo-Hermoso, Rachael E. Tarlinton

**Affiliations:** 1 School of Veterinary Medicine and Science, University of Nottingham, Sutton Bonington, Loughborough, United Kingdom; 2 Epidemiology and Environmental Health Group, Department of Infectious Animal Diseases and Global Health, Animal Health Research Centre, National Centre Institute for Agriculture and Food Research and Technology, Spanish National Research Council (CISA-INIA-CSIC), Madrid, Spain; 3 Departamento de Ciencias Biológicas y Biodiversidad, Universidad de Los Lagos, Av. Fuchslocher 1305, 5311157 Osorno, Chile; 4 Institute of Ecology and Biodiversity, Victoria 631, 4070374 Concepción, Chile; 5 Cape Horn International Center, O’Higgins 310, 6350000 Puerto Williams, Chile; 6 Centro de Conservación de la Biodiversidad Chiloe Silvestre, Ancud, Chile Chiloe Island

**Keywords:** Feline leukemia virus, domestic cats, NGS, wild felid, illumina

## Abstract

Feline leukemia virus (FeLV) is widespread in domestic cats and frequently spills over into wild felid populations, causing severe outbreaks in nondomestic felid populations. Domestic cats carry both endogenous FeLV (enFeLV) and exogenous variants of FeLV (exFeLV), which can recombine to generate novel variants with alternate receptor usage. In contrast, most nondomestic felids, including the guigna, do not harbor enFeLV. This study applied amplification of the FeLV envelope gene combined with Illumina sequencing to characterize FeLV envelope gene diversity and transmission dynamics in guignas (*Leopardus guigna*), a small wild felid native to Chile and Argentina. Seven polymerase chain reaction (PCR )amplicons from five free-ranging guignas were sequenced. Illumina sequencing was successfully conducted, and subsequent phylogenetic analysis indicated that the detected infections were most closely related to strains circulating in Chilean domestic cats. However, a distinct guigna-specific cluster with unique sequence variations was identified, suggesting that FeLV transmission also occurs among guignas independent of domestic cats. Phylogenetic analysis showed that all guigna sequences formed a Chile-specific FeLV clade, closely related to domestic cat viruses but clearly separated from international FeLV-A strains.

**Supplementary Information:**

The online version contains supplementary material available at 10.1186/s13567-026-01770-6.

## Introduction

Feline leukemia virus (FeLV) is an enveloped retrovirus that belongs to the family *Retroviridae*, genus *Gammaretrovirus*. The FeLV genome consists of a homodimer of single-stranded, positive-sense RNA, each strand of approximately 8.4 kb in length. It encompasses three genes: *gag* (encoding matrix, capsid, and nucleocapsid proteins), *pol* (encoding reverse transcriptase, protease, and integrase), and *env* (encoding envelope proteins, including surface and transmembrane subunit). These are flanked, 5′ and 3′, by two identical untranslated regulatory sequences called long terminal repeats (LTRs) [1]. During infection, the RNA genome is copied into a DNA genome by the reverse transcriptase enzyme and is inserted as a provirus into the host genome, leading to lifelong infection, and facilitates recombination events to contribute to the FeLV diversity [2].

FeLV has a worldwide distribution in domestic cat populations and is one of the most important pathogens for their health [3]. The virus can replicate in many tissues, the most important being lymphoid tissues, salivary glands, and intestinal tracts [4]. It causes clinical signs such as immune and bone marrow suppression, lymphadenopathy, lymphoma, leukemia, oral lesions, and respiratory diseases [5]. The virus is excreted in saliva, urine, feces, milk, and nasal secretions [6]. Social activities such as grooming, sharing food and water dishes, nursing, and bites during fights are the main transmission routes. It can also be transmitted via blood contact (blood transfusion or contaminated instruments) or sharing of litter trays [7]. Another important route of transmission is vertical transmission, where the queen cat spreads the virus to her neonatal kittens, either transplacentally, during parturition, or during nursing of kittens [8]. FeLV also has importance in wild felid health and conservation, causing severe disease and mortality in genetically bottle-necked populations such as the Iberian lynx (*Lynx pardinus*) [9] and is notably harmful in the Florida panther (*Puma concolor coryi*) [10, 11]. Although, the virus can persist in wild species, domestic cats have been the infection source for nondomestic animals in the majority of cases, including Iberian lynx [9], Florida panthers [12], and both pumas and bobcats [13]. Thus, FeLV represents a critical pathogen at the domestic–wildlife interface.

However, endogenous FeLV (enFeLV) are retroviruses that have integrated over millions of years into the cat genome. EnFeLV were acquired during the divergence of the Felidae and are present in domestic cat chromosomes and their close relatives in the *Felis* genus. They are transmitted from parents to descendants through chromosomes [14]. The presence of enFeLV can result in recombination events with exogenous FeLV (exFeLV) and produce FeLV-B, a more virulent variant with altered cellular tropism [2]. Because nondomestic felids (except *Felis silvestris*) lack enFeLV, their FeLV infections provide a unique opportunity to study exogenous viral evolution without interference from endogenous elements [14].

In domestic cats, FeLV-A is the most widespread group that can be horizontally transmitted and is often described as the least pathogenic subtype [15, 16]. All other groups arise de novo from FeLV-A mutation or by recombination with endogenous elements [3]. FeLV-B is the second most common subgroup, being detected in roughly half of all FeLV-A cases [17]. Overall, FeLV-B is associated with worse clinical symptomatology and prognosis [15]. Although FeLV-B has historically been described as unable to be horizontally transmitted without FeLV-A, studies in domestic cats may suggest that FeLV-B transmission may occur [18, 19]. In particular, a more robust case was documented in a puma infected by FeLV-B, without FeLV-A; these subgroup dynamics highlight the evolutionary flexibility of FeLV and the importance of studying its genetic variability across domestic and nondomestic species [10].

The majority of these variations occur in the *env* gene, which is the binding point between the virus and host cell [20]. The *env* gene is composed of the surface protein (SU) and transmembrane protein (TM). SU includes three regions from 5′ to 3′: receptor-binding domain (RBD), proline rich region (PRR), and C-domain. The RBD and C-domain are involved in cellular tropism; therefore, changes in these regions can modify cell receptor usage and thereby change clinical progression [20, 21]. More specifically, there are three main variable regions (VRA, VRB, and VRC) identified, along with specific amino acid changes that are associated with changes in receptor usage [22].

FeLV epidemiology in domestic cats is related to multiple factors. In developed countries, FeLV is generally controlled, showing a prevalence between 0.7% and 5.5% in Europe [23] and 3.1% FeLV in North America [24]. Conversely, the situation in South America is highly variable, ranging from 3% in north-eastern Brazil [25] to 59.44% in Colombia [26], while in Chile, the prevalence has been described to range from 20.2% to 33% in rural areas [27, 28] and 14% to 54.5% in cities [29, 30]. This marked heterogeneity reflects differences in cat management, human population density, and veterinary care access across the continent and within Chile.

In South American nondomestic species, molecular detection of FeLV has been reported in guignas (*Leopardus guigna*) from Chile [27, 28], ocelots (*Leopardus pardalis*) from Ecuador [31], and multiple species from Brazil, including ocelots, oncillas (*Leopardus tigrinus*), jaguarundis (*Puma yagouaroundi*) [32], and jaguars (*Panthera onca*) [33, 34]. In North America, FeLV has been associated with clinical signs and pathological findings in a captive bobcat (*Lynx rufus*) in the USA [35], as well as outbreaks in Florida panthers (*Puma concolor coryi*) and some cases in bobcats [10–13]. These repeated spillover events have demonstrated the capacity of FeLV to cross species barriers and establish sustained transmission in susceptible wild felids.

The Florida panther and Iberian Lynx (*Lynx pardinus*) have been the most emblematic examples of FeLV impact in nondomestic felids. The worst consequence of FeLV introduction in these species has been the exacerbation of the consequences of genetic bottlenecks, causing population size reductions in already vulnerable populations [9–12, 36, 37]. The most recent FeLV survey in North America demonstrated a prevalence of 3.12% in pumas, 0.47% of bobcats, and 6.25% in domestic cats. A total of 20 transmission events were inferred, 3 domestic cats to puma spillovers, 3 confirmed puma-to-puma transmissions, and 14 events likely to have originated from domestic cats [13]. The Iberian lynx has also been severely affected by FeLV [38]. Between 2003 and 2007, at the beginning of the outbreak, 21% of animals tested positive for FeLV, and 6 individuals died from FeLV-related disease [39]. As a consequence, intensive management strategies have been implemented in both species, including vaccination campaigns, isolation of progressively infected animals, and translocations to decrease the inbreeding rates and disease risk [10, 11, 36, 37]. After more than a decade of intervention, the epidemiological situation has shifted from acute mortality [9, 11, 12] to more controlled scenarios that allow continuous surveillance. This has deepened the understanding of FeLV dynamics in endangered species. However, changes in population density and reduced habitat availability have led to greater overlapping areas and higher tolerance of intraspecies interactions, facilitating, for example, puma-to-puma transmission [10, 11, 13, 40]. Despite this, domestic cats remain the most likely source of initial infection [10, 13, 39], although there is a risk that some viral variants may become more adapted to different species such as FeLV-B in pumas [10]. Additionally, reports of enteritis associated with FeLV have been described [32, 41], expanding the traditional profile of FeLV as the cause of tumoral and immunosuppressive disease, expanding the knowledge about how FeLV can severely affect endangered felids and highlight the importance of early detection and molecular monitoring.

The guigna has been classified as vulnerable for over a decade, with a continually decreasing population trend due to severe fragmentation of its habitat. It has recently been downlisted to least concern due to better information available on the species, though the same threats are still present across its range [42, 43]. Similar to other felids, guignas are solitary animals, except during mating [44]. Reduced genetic diversity has been correlated with human-dominated landscapes [42], as well as pathogen infections associated with the presence of humans and domestic animals, such as canine parvovirus, feline immunodeficiency virus, and FeLV [27, 45, 46]. In contrast to the managed FeLV outbreaks in the USA and Spain, guignas in Chile have shown even higher FeLV prevalence (>20%) than observed in Florida panthers or Iberian lynxes [28, 45]. This could reflect increased interactions with domestic cats due to habitat disruption, or more frequent guigna-to-guigna contact. Moreover, FeLV remains uncontrolled among domestic cats in Chile [29], similarly to other Latin American countries [26, 47, 48]. Despite the high prevalence and conservation relevance of FeLV in guignas, no studies to date have characterized full *env* gene diversity, intrahost variation, or phylogenetic structure using next-generation sequencing.

On the basis of this information, we conducted a molecular survey for FeLV infection in free-ranging guignas using targeted *env* amplification followed by Illumina sequencing to characterize viral diversity and transmission patterns. Our objective was to determine the genetic diversity of FeLV circulating in guignas and identify the likely source of infection by comparison with domestic cat sequences. We hypothesize that FeLV infections in guignas originate primarily from domestic cats, although subsequent guigna–guigna transmission may have also been occurring, leading to distinct viral variants and unique intrahost mutation patterns. Therefore, we expected to find (i) phylogenetic clustering of guigna FeLV sequences with Chilean domestic cat FeLV-A strains and (ii) guigna-associated cluster/s with nucleotide and amino acid substitutions suggestive of viral divergence within the species.

## Materials and methods

### Samples and end-point PCR

Free-range guigna samples were sourced from a previous study detecting FeLV using PCR based on LTR regions [27]. These animals were sampled with permission from the Chilean Agriculture and Livestock Service and approved by the Animal Ethics Committee (Institute of Ecology and Biodiversity). The samples were processed at the Universidad de Los Lagos, Osorno, Chile. In all cases, DNA extraction was performed using the commercial QIAGEN DNeasy Blood and Tissue kit following the manufacturer’s instructions (Qiagen, Valencia, California, USA).

**Figure 1:**
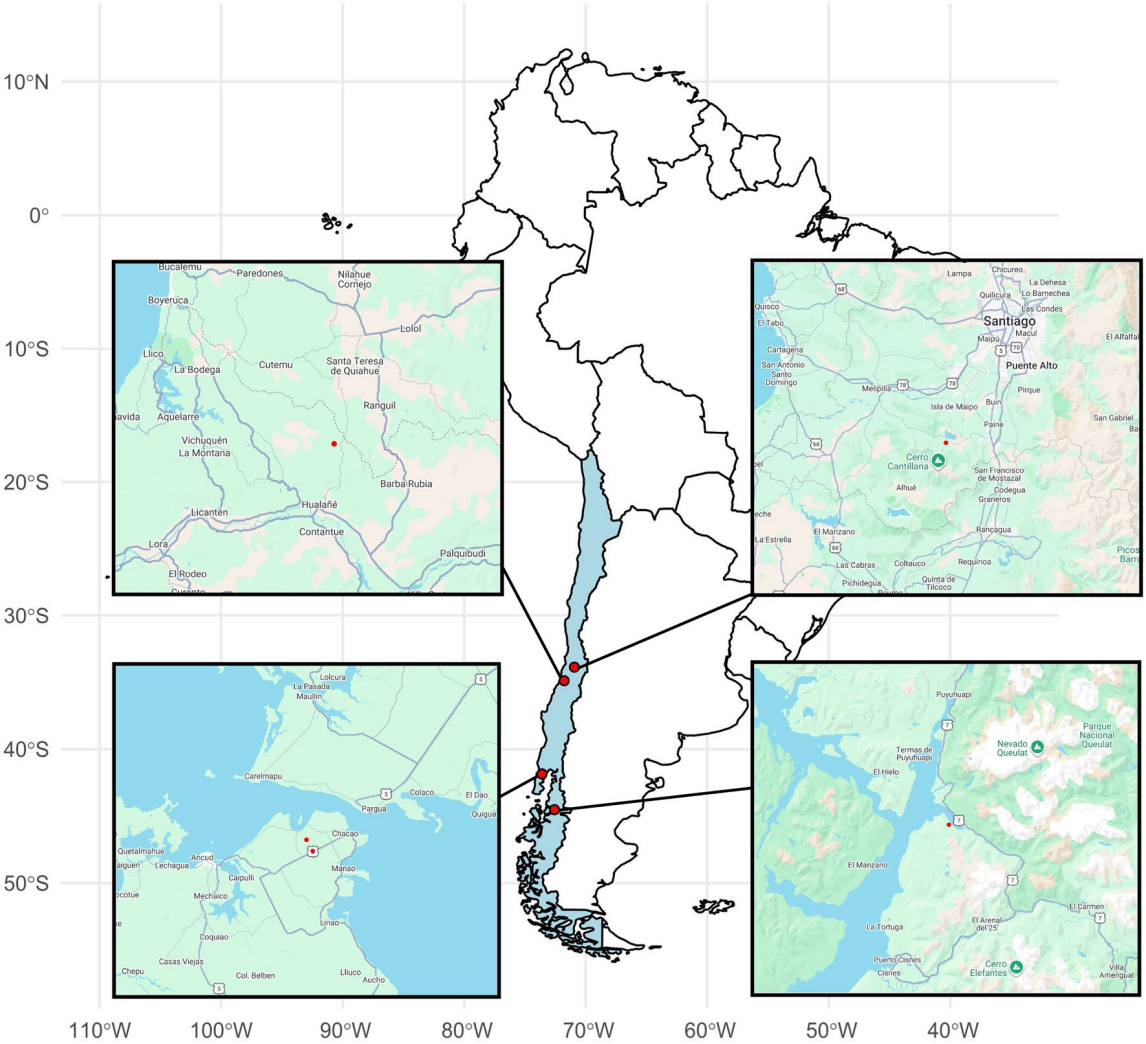
**Map of South America showing the geographical location of Chile (light blue)**. Small circles indicate the four sampling sites of samples sequenced. One individual was sampled near Hualañe, Paine, and Puerto Cisne, whereas two were sampled near Ancud.

All guigna samples sequenced were from males captured in rural areas of central and southern Chile (Figure 1). These locations were Puerto Cisnes (44° 31′ 25.68″ S, 72° 34′ 12.18″ W), Paine (33° 52′ 21.71″ S, 70° 55′ 56.30″ W), Hualañe (34° 53′ 21.86″ S, 71° 46′ 21.92″ W), and two locations in Ancud (41° 50′ 25.21″ S, 73° 36′ 20.98″ W; 41° 51′ 27.73″ S, 73° 35′ 34.03″ W).

End-point PCR using two primer pairs following methods from Erbeck et al. [49] was used to amplify the envelope gene hypervariable region and 3′LTR region from FeLV-A and FeLV-B. PCR was performed using GoTaq^®^ Long PCR Master Mix (Promega), and each reaction consisted of: 12.5 µL of GoTaq^®^ Long PCR Master Mix; 0.5 µL (10 µM) forward primer; 0.5 µL (10 µM) reverse primer; 9.5 µL of nuclease-free water; and 2 µL of template DNA to reach a final volume of 25 µL per reaction.

The same protocol was used for each pair of primers. Thermocycler conditions used followed the Erbeck et al. [49] protocol, with some modifications according to the polymerase requirements. The protocol was: initial denaturation at 94 °C for 2 min followed by 45 cycles of denaturation at 95 °C for 30 s; annealing at 63 °C for 30 s; extension at 72 °C for 2 min; and a final extension at 72 °C at 2 min. All PCR runs included extraction blanks and nontemplate controls to monitor contamination. The thermocycler conditions were tested with a gradient PCR to select the optimal annealing temperature, and every set of PCR reactions included a negative control with no template (DNA-free water) and a positive control (domestic cat FeLV-A DNA). The PCR products were run by electrophoresis on a 1% agarose TAE (Fisher Scientific^®^) gel at 80 V, 400 mA for 60 min. The Nucleospin^®^ Extract II kit (Macherey–Nagel^®^) was used for DNA purification, according to the manufacturer’s instructions.

DNA quantification was performed with a Qubit 4 Fluorometer (Thermo Fisher Scientific^®^). FeLV-A and FeLV-B amplicons of enough quality and with minimum DNA quantity of 1.1 µg per sample were selected for Illumina sequencing.

### Genome sequencing and read processing

Amplicons from *env* genes were amplified and sequenced by the Illumina platform (HiSeq4000) with 300 base pair (bp) paired-end distances using a coverage of 30× by Novogene Europe, Cambridge, UK. Library preparation followed Novogene standard protocol for amplicon sequencing.

Illumina reads were trimmed and adaptors removed using FastP version 0.23.1 [50] with default stringency parameters. De novo assembly from Illumina reads was performed using MEGAHIT [51]. The contigs generated by MEGAHIT were mapped to FeLV-A FAIDS (M18247) using Bowtie2 [52]. All contigs were extracted and imported to Geneious version 2023.0.4 (Biomatters Inc., Newark, NJ, USA) to annotate and continue phylogenetic analysis. The dataset was constructed in Geneious using all available complete *env* gene FeLV-related sequences, and they were annotated and aligned with MAFFT [53].

Data are available in GenBank under BioProject no. PRJNA1328684. The corresponding SRA files are available using the following accession nos. Lpgui_FeLV_1a (SRR35412604); Lpgui_FeLV_1b (SRR35412603); Lpgui_FeLV_2a (SRR35412602); Lpgui_FeLV_2b (SRR35412601); Lpgui_FeLV_3 (SRR35412600); Lpgui_FeLV_4 (SRR35412599); and Lpgui_FeLV_5 (SRR35412598).

The alignment was sent to the IQTREE web server [54], where a maximum likelihood tree was inferred using the ModelFinder [55] to select the best-fit model nucleotide substitution. The model chosen was the general time-reversible model (TIM2 + F + G4), and branch support analysis was ultrafast with 1000 bootstrap replicates [56]. EnFeLV was used as an outgroup to root the tree, and the phylogenetic tree was visualized in FigTree version 1.4.4 [57].

### Intrahost variation analysis and recombination analysis

The intrahost single-nucleotide variation (iSNV) analysis was carried out using BWA-MEM [58] and iVar [59]. Whole libraries were mapped to the FeLV-A sequence (M18247) using BWA-MEM, and consensus sequences were generated using the iVar consensus command for each library. All reference sequences generated by iVar were cut at nucleotide 66 (M18247) to initiate at the same nucleotide position. The output was used to call single-nucleotide variants and indels in iVar. The minimum base quality parameter was 10 (*Q* > 10), and only variants with frequencies above the 1% cutoff (*p* < 0.05) were retained**.**

## Results

A total of 20 guigna samples were available for PCR; of these, 9 animals were positive using FeLV-A primers, and 8 were positive for FeLV-B primers. The *env* gene was successfully sequenced from five guignas, corresponding to five FeLV-A amplicons and two FeLV-B amplicons. All resulting contigs were approximately ~1.9 kb in length. The FeLV-A PCR amplicons matched the expected amplicon size (~1.9 kb) and did not show the insertions or deletions commonly found in other variants of FeLV.

Although FeLV-B specific primers yielded amplicons, they revealed only FeLV-A sequences with random nucleotides attached to the 5′ end. The FeLV-A contigs shared 97.2–100% nucleotide identity among themselves and 98.8–100% identity at amino acid level. When compared with FeLV sequences from Chilean domestic cats [60], the guigna sequences showed 97.2–99% nucleotide identity and 95.6–99.7% amino acid identity.

BLASTn analysis revealed that the guigna sequences shared 98.86% identity with FeLV-FAIDS strain (GenBank accession no. M18247.1), and 98.44% identity with FeLV_US_x2653_Fca2018 from the USA (MH116004.1). The FeLV-B amplicons demonstrated a similar identity percentage, although with a reduced query coverage due to random nucleotides attached to the 5′ end.

**Figure 2:**
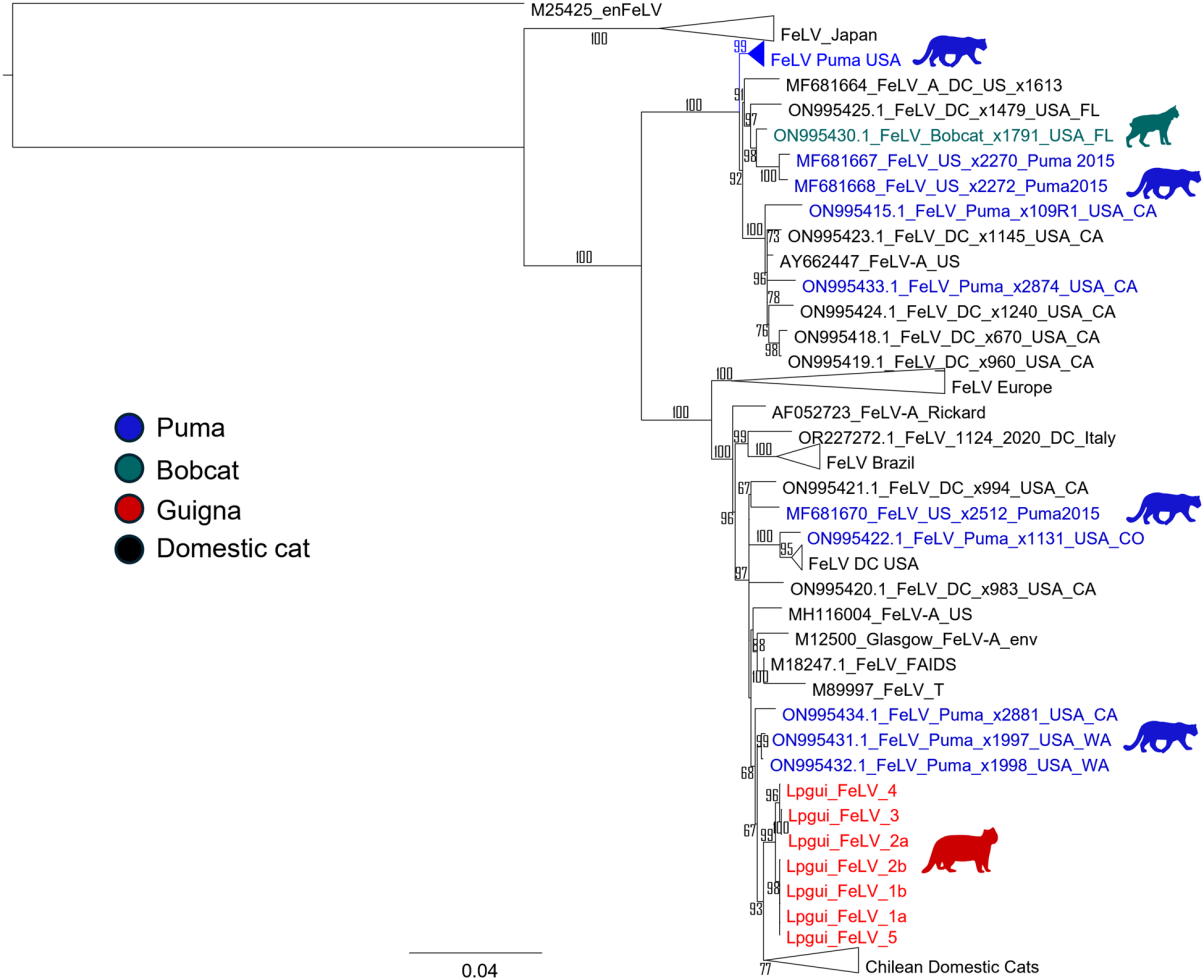
**Phylogenetic tree of the FeLV env gene (nucleotide sequence) was constructed using 1000 bootstrap approximations and rooted against enFeLV (M25425)**. The analysis includes only FeLV-A sequences from domestic and nondomestic felids, including all previously reported Chilean sequences. Clades from Chilean domestic cats and FeLV from Europe, Brazil, Japan, and Brazil have been collapsed for clarity (horizontal triangles). Guigna sequences are shown in red and form a distinct cluster adjacent to the Chilean domestic cat clade. Puma sequences are shown in purple and the bobcat sequence is shown in turquoise. Silhouettes have been adapted from Phylopic and guigna from Palomo-Munoz (CC BY 4.0).

Contigs assembled using MEGAHIT were aligned with FeLV-A sequences from domestic cats in Europe, the USA, Japan, Brazil, and Chile, as well as with sequences from nondomestic felids such as pumas and Iberian lynx, when available. Guigna samples clustered together in a robust phylogenetic separation (>90% of bootstrap support), without distinction between FeLV-A and FeLV-B amplicons (Figure 2). This guigna clade is adjacent to the cluster of Chilean domestic cats (Figure 2 and Additional file 1) and was positioned near a puma sequence from the USA. All were located inside a bigger cluster that includes the FeLV-FAIDS (M18147.1) and Rickard (AF052723) reference strains.

**Figure 3:**
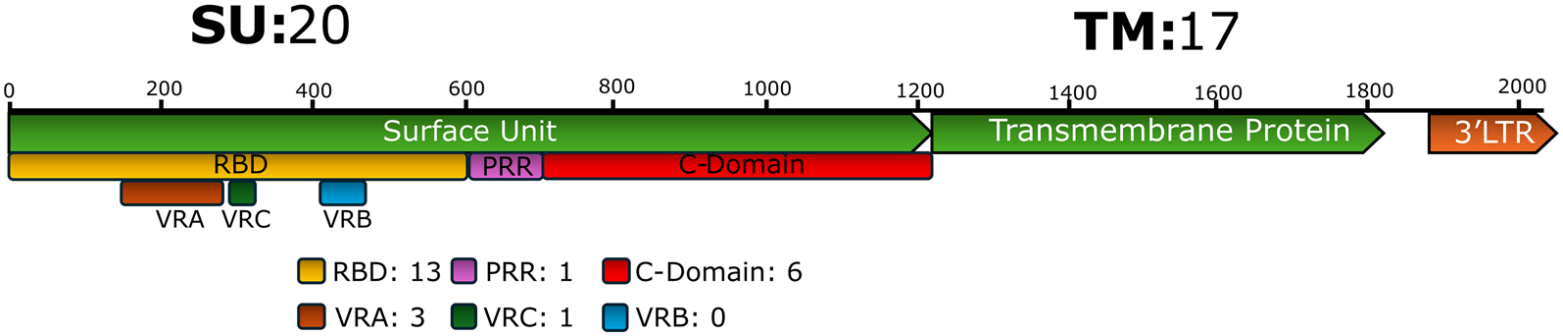
**Genome structure amplification scheme showing unique iSNVs across the seven amplicons from five guignas**. Overall, 20 events occurred in the surface unit and 17 occurred in the transmembrane protein.

**Figure 4:**
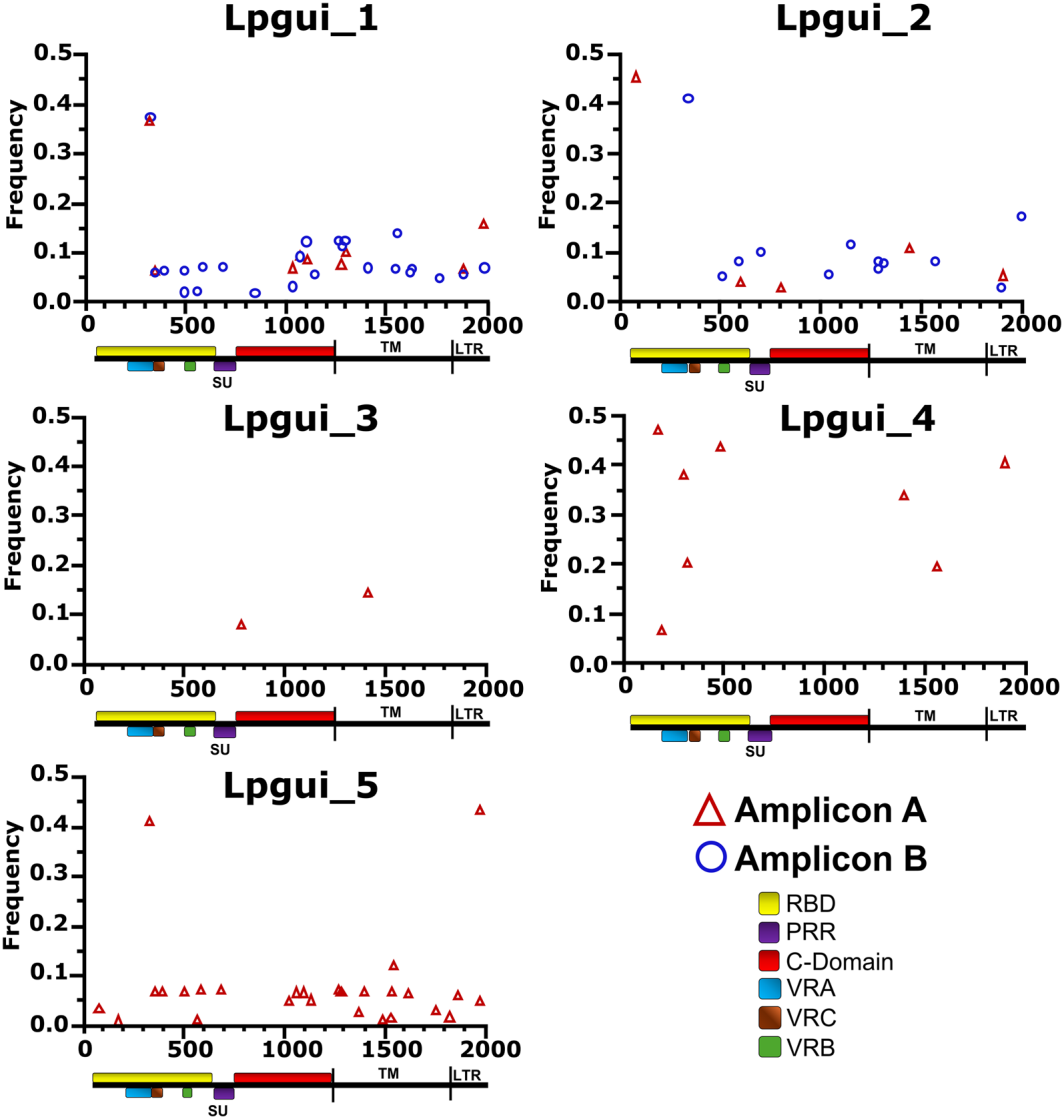
**iSNV sites detected in env gene amplicons from Illumina sequencing.** iVar software was used to generate a consensus sequence per library and as a reference sequence (~1.8 kb). Blue circles represent iSNV obtained using primers to amplify FeLV-B, and red triangles represent iSNV obtained with FeLV-A primers. Each graph represents a different animal.

The iVar analysis identified a total of 96 intrahost single-nucleotide variants (iSNVs), corresponding to 43 unique nucleotide positions across the seven amplicons obtained from five guigna individuals (Figure 3). Overall iSNV counts per animal ranged from 2 to 52, indicating strong interindividual heterogeneity. Among them, Lpgui_FeLV_1b and Lpgui_FeLV_2b showed the highest number of iSNVs with substantial variation also observed in sample Lgui_FeLV_5. In contrast, Lpgui_FeLV_3 presented only two iSNVs, while Lpgui_FeLV_4 showed eight iSNVs but with comparatively higher variant frequencies compared with the other animals (Figure 4).

In terms of genomic distribution, the majority of iSNVs (50) were in the SU region, followed by 34 positions within the TM region. Only seven iSNVs were identified in the LTR region. Considering only unique iSNVs across all sequences, there were 20 sites across the SU, 17 sites across the TM, 2 in noncoding areas, and 5 in the LTR. The proportion of TM mutations (38.6%) was higher than typically reported in domestic cats, where TM is comparatively conserved. Within the SU domain, 31 iSNVs were identified in the RBD, 3 in the PRR, and 16 in the C-domain. As expected, the RBD was the most variable segment of the *env* gene.

**Figure 5:**
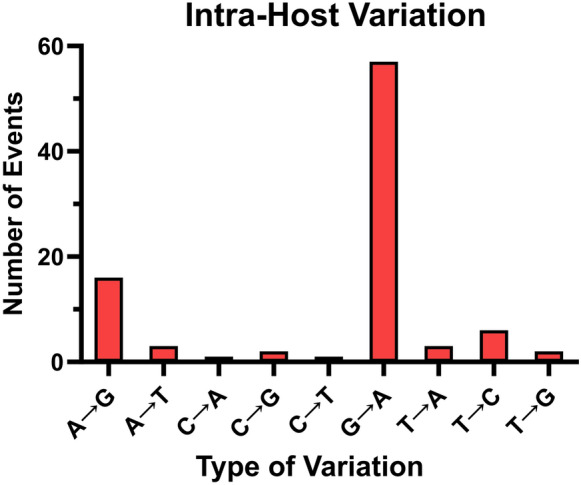
**Bar graph of nucleotide variation in sequences obtained from Illumina sequencing for guigna samples.**

Analysis of the type of iSNVs showed that G → A transitions were the most common, with a total of 57 events (62.6%). Other types of substitutions included 16 A → G (17.6%), 3 A → T and 3 T → A (3.3%), 2 C → G (2.2%), C → A and C → T had 1 event each (1.1%), 6 events of T → C 6 (6.6%), and 2 T → G (2.2%) (Figure 5).

Out of the total 96 iSNVs, 84 were located within coding regions, with 20 synonymous and 64 nonsynonymous mutations. These nonsynonymous variants were detected at different frequencies across samples, involving 26 distinct amino acid positions. Of these, 15 were G → A transitions, with 13 of them strongly suggestive of APOBEC mediation, based on typical APOBEC motifs (AGG or GGG). Only one was less likely to be an APOBEC substitution (GAA to AAA).

Nonsynonymous iSNVs were found in both the SU and TM regions, affecting eight positions in SU and seven in TM. The most frequently substituted amino acid was glycine (Gly) replaced in eight positions by glutamic acid (Glu), arginine (Arg), or serine (Ser). One notable iSNV at position 1620nt introduced a premature stop codon, potentially truncating the envelope protein and impacting viral function.

Based on the de novo assembled contigs, guigna samples were more conserved at the amino acid level compared with Chilean domestic cat sequences. Four notable amino acid variations were identified differing between the Chilean domestic cats and other reference sequences (Table 1). At position 59 of the *env* gene, within the VRA, guigna sequences showed a serine (Ser), whereas Chilean cats presented either aspartic acid (Asp) or asparagine (Asn) at the same position. At position 60, three guigna contigs exhibited Ser, while four contigs showed proline (Pro), in contrast to domestic cats, which consistently had Pro at this position. Afterward, at position 71, all Chilean domestic cats and guignas showed aspartic acid (Asp) compared with reference sequences. Position 165, however, showed only variation in guignas, carrying either arginine (Arg) or lysine (Lys). Notably, position 249 consistently showed leucine (Leu) across all Chilean sequences (domestic cats and guignas), unique to the Chilean cluster and not identified in other sequences from GenBank.

**Table 1:** **Amino acid variation identified across guigna and domestic cats**

Amino acid position	59	60	71	165	249
DC_Chile	D	P	D	R/K	L
Lpgui_1a	S	P	D	R	L
Lpgui_1b	S	P	D	R	L
Lpgui_2a	S	P	D	R	L
Lpgui_2b	S	S	D	K	L
Lpgui_5	S	S	D	K	L
Lpgui_4	S	S	D	K	L
Lpgui_3	S	S	D	K	L
FAIDS (M18247)	S	P	S	K	P
Rickard (NC_001940)	D	P	S	R	P
Glasgow (M12500)	N	P	S	R	P
Iberian lynx (EU293175-94)	D	P	S	R	P
Puma US (ON995415-34)	D/S	P	S	R/K	P

*DC_Chile: Chilean cluster of domestic cats from Castillo et al. [59].

Several positions were recurrent across individuals: positions 325 (Gly → Arg) and 1269 (Met → Ile) were present in five samples, while positions 494 (synonymous), 1027 (Ala → Gly), 1269 (Met → Ile), 1276 (Gly → Arg), 1291 (Gly → Ser), and 1879 (noncoding area) were found in four samples. Notably, the common nucleotide variants identified in the de novo assembly were also detected in other samples at lower frequencies, confirming common variant sites within the population.

For example, the amino acid substitution at position 60 (Ser → Pro) was observed in sample Lpgui_FeLV_4 at a frequency of 47%, and in other samples at lower frequencies (<10%). Similarly, that at position 169 (synonymous) was found at 43% in Lpgui_FeLV_4, and at 7% in both Lpgui_FeLV_2b and Lpgui_FeLV_5. Overall, the frequency of iSNVs was between 47% and 1% of total reads.

## Discussion

The findings described in this study extend previous knowledge regarding the FeLV situation in guignas. Our results confirm that domestic cats are the primary source of FeLV infection for guignas, with very close clustering of Chilean domestic cat and guigna sequences indicating region-specific circulation of a Chilean FeLV-A lineage. We also identified a distinct guigna-specific FeLV cluster, characterized by unique mutations not observed in domestic cats. It is possible that very rapid adaption of virus to guigna is occurring, as occurs for avian flu isolates in mammals [61], but the complete separation of the guigna and domestic cat sequences in our study suggests that, while the virus originally arose from Chilean domestic cats, ongoing guigna-to-guigna transmission is now occurring. The receptor usage strongly influences host range and pathogenicity, in consequence, *env* gene sequencing is essential for understanding these cross-species transmission events.

Previous studies assessing infection prevalence across large regions of Chile have relied on 3′LTR diagnostic PCR [27, 28]. This method is more sensitive considering that it is a smaller amplicon (211 bp), and it is also highly conserved, decreasing false-negative results [62]. These characteristics make it suitable for prevalence calculations. However, analysis of the envelope gene, which is a hypervariable gene, provides additional information about the genetic dynamics of the virus [10, 13, 63], including receptor usage for cell entry and potential clinical progression [20, 64], although the high similarity and recombination events between enFeLV and FeLV-A reduce the specificity of these primers, as we observed with our FeLV-B primers amplifying FeLV-A amplicons, and as previously described [63, 65].

In the USA, FeLV in pumas has demonstrated multiple introduction events from domestic cats to pumas, followed by puma-to-puma transmission [10, 13]. Two distinct FeLV-A clades were identified, resembling the situation in Japan where multiple FeLV-A clusters coexist [13, 63]. The epidemiological situation in pumas and Iberian lynx differs markedly from the guigna context. Both Iberian lynx and North American pumas are apex predators, frequently preying on domestic cats [12, 66, 67]. In contrast, the guigna is the smallest wild felid in the American continent, half the size (2 kg) of a domestic cat, with less frequent direct interactions [68]. Contact between domestic cats and guigna occurs mainly at forest edges near rural human settlements, where guigna may enter human areas when hunting poultry [69, 70], and/or rural free-roaming domestic cats can move away from their households up to 2.5 km into forested areas [71], potentially leading to aggressive domestic cat–guigna encounters [28]. This behavioral context also likely explains the higher prevalence in male guigna and Iberian lynx [9, 27, 37]. Following infection, males can transmit the virus to females during mating, as guigna, similar to most felids, are solitary outside of mating periods [44].

The phylogenetic tree presented in this study incorporated sequences previously described from eight domestic cats sampled in Concepcion, Chile [60], a city located between the northern (397 km to the north) and southern sampling sites (886 km to the south), as well as samples from animals from diverse worldwide geographic origins and two other wild felid species. In addition, it used three different sequencing methods. A distinct Chilean cluster was clearly identified when compared with reference sequences from Europe and the USA [13, 20, 65]. Within this cluster, all Chilean sequences form a terminal cluster with a specific subcluster for guigna sequences. Interestingly, none of the Chilean sequences were related to the Brazilian strains, consistent with observations from the LTR-*gag* PCR analysis, demonstrating the circulation of distinct FeLV-A strains across South America.

As a first overview, the absence of large deletions or insertions in *env* suggests that these viruses may not be highly pathogenic variants such as FeLV-C, FeLV-T, or FeLV-B strains [64, 72]. Among the Chilean sequences, two amino acid changes were uniquely identified in guignas at positions 60 and 61, within the VRA region, which is critical for receptor interaction [20, 64, 72]. The presence of Ser at position 60 has been described previously in Iberian lynx [73], some pumas [13], and domestic cats with tumors [64]. However, Ser at position 61 has not been previously described. While the presence of amino acid combinations of Asp52 and Asn60Asp mutations are known to enhance receptor binding, viral entry, and viral replication [19, 74], the significance of Ser at this position is unknown. These unique variations may represent specific viral adaptations to guigna receptors, and comparative studies on the sequence and structure of guigna FeLV receptors could elucidate the virus infectivity and replication efficiency in this wild felid. Another notable amino acid substitution, Pro249Leu, was observed exclusively in all Chilean FeLV strains, whether from wild or domestic felids, and appears to be a feature of the currently circulating Chilean strains.

Evidence from Iberian lynx suggests that FeLV infection in this species is typically in a regressive state, with viral replication largely controlled, except in a few progressively infected individuals [37]. This may partly explain the challenges in amplifying longer viral fragments observed here and in previous studies [27, 41]. A similar situation was described in a lynx in Germany, after an attempt to sequence the whole genome of FeLV using next-generation sequencing (NGS) yielded only partial sequences, requiring re-amplification with the Sanger method to complete the genome sequencing. This sequence showed notable differences regarding published reports, but there is no evidence of FeLV in other lynxes from that area [41]. Studies recorded FeLV PCR-positive guignas showing very low frequency of clinical signs, no histopathological evidence of disease, and negative serology, all suggestive of regressive infection [45]. Although we cannot definitively conclude that guigna experience similar regressive infections, the low diversity of FeLV strains in our samples and the low variation within each host suggest limited active replication and few new introductions of virus into the guigna population.

A notable finding was the unusually high proportion of iSNVs in guignas that occurred in the TM region of the *env* gene, resulting in amino acid variations and even premature stop codons. In domestic cats, in contrast, variation is typically concentrated in the SU region, especially the RBD area, while the TM domain remains highly conserved, even between FeLV-A, FeLV-B, and other recombinants [20].

A plausible explanation for the unexpected higher ratio of TM iSNVs is pressure from the guigna APOBEC antiviral system. APOBEC3 enzymes preferentially deaminate cytosines in AGG and GGG motifs, likely causing G → A in GG/GC or GA dinucleotide, generating higher numbers of hypermutations that produce noninfective virions [75, 76]. Although APOBEC has a stronger impact on FIV and FFV than on FeLV in domestic cats [77], its activity may still drive the unique variation patterns we observe in guignas. A previous study using the Roche 454 sequencing platform to sequence the SU gene in Iberian lynx infections reported some effects of the APOBEC system. They identified 25 sites of variations in SU, nearly half of which (48%) led to nonsynonymous changes, being predominantly Trp → stop codon and Gly → Arg [73]. By contrast, our analysis additionally included TM protein and also indicated Gly as a frequently replaced amino acid. We identified a higher level of nonsynonymous variations (62.6%), and a sole premature stop codon was identified within the TM protein. The Iberian lynx study sampled fewer animals and detected fewer iSNVs per animal, while in our dataset, one guigna harbored only 2 iSNVs and another showed over 50 different positions. Therefore, individual and species effects may also influence the efficacy of the APOBEC3 system in FeLV infections.

Comparative phylogenetic analyses of felid APOBEC systems indicate that the domestic cat and puma systems are closely related, whereas the lynx APOBEC diverged slightly earlier [77]. Specifically, puma and domestic cat lineages diverged ~6.7 Mya, followed by lynx lineage divergence ~7.2 Mya [78]. The *Leopardus* lineage (including guigna) diverged over 8 Mya [78, 79]. This evolutionary distance could indicate that guigna APOBEC3 may interact differently with FeLV genomes compared with domestic cat APOBEC3. Although it cannot be directly compared, FIV from pumas was less effective at infecting domestic cats, because the virus was less adapted to a different species and in consequence was more susceptible to domestic cat APOBEC [77]. These findings support the idea that FeLV from domestic cats may likewise be poorly adapted to guigna APOBEC3, resulting in increased viral hypermutation in guignas, although substantial experimental work may be required to confirm this hypothesis.

When our iSNVs were mapped against published FeLV sequences, most appeared to be random, but several matched mutations documented in other hosts. For instance, the Gly109Arg substitution was originally described in domestic cats with tumors [64]. The Val262Ile variation has been described in cats suffering tumors and in Brazilian cats [20, 64]. Lys343Glu has been described across a wide range of hosts, including cats from the USA, an Iberian lynx, European cats, the Rickard reference strain, pumas, and Brazilian cats [13, 20, 73, 80]; Ala469Thr and Ala513Thr substitutions have been previously observed in US pumas [10]; and the Glu542Lys mutation has been reported in a US domestic cat [18].

Only two other studies have used NGS to characterize FeLV in nondomestic felids, one case report and one using one of the first NGS methods, which limits direct comparisons with our dataset [41, 73]. Nevertheless, we have successfully described *env* variation in guignas using Illumina sequencing. Given that receptor usage strongly influences host range and pathogenicity, *env* sequencing is essential for understanding cross-species transmission events. Another key advantage was the absence of enFeLV in guigna genomes, which reduced the interference of incorrect amplification and bioinformatic artifacts that were observed in domestic cat analysis [60]. The Illumina method proved highly effective for variant detection and produced results broadly consistent with those from domestic cats using the same method.

Rapid human migration from urban areas into Chilean native forest is driving significant landscape changes [81]. As contact between domestic cats and other nondomestic felids increases, pathogen spillover risk will continuously increase. Additionally, this study provides evidence that there is likely guigna-to-guigna transmission occurring and highlights the need to monitor clinical impacts in this vulnerable species. It is therefore critical to maintain ongoing surveillance of FeLV both in domestic cats and in guigna populations. Intensive management strategies have been successfully implemented in both North American pumas and Spanish lynxes, including vaccination campaigns, isolation of progressively infected animals, and translocations to decrease the inbreeding rates and disease risk [10, 11, 36, 37], which may provide guidelines to establish measures for disease control. The effectiveness of commercial domestic cat FeLV vaccines in controlling diseases has been widely demonstrated in Iberian lynx and North American puma species recovery programs and will be useful to establish in potential future guigna programs.

## Supplementary Information

**Additional file 1. Uncollapsed phylogenetic tree of the FeLV env genewas constructed using 1000 bootstrap approximations and rooted against enFeLV**. The analysis includes only FeLV-A sequences from domestic and nondomestic felids, including all previously reported Chilean sequences. Guigna sequences are shown in red. Puma sequences are shown in blue, and bobcat sequence is shown in turquoise.

## Data Availability

All data supporting the findings of this study are included in this published article and have been deposited in the NCBI GenBank database.
