# Supplementary figures and images for: Cross-species transmission of host adaptation of feline leukemia virus between domestic cats and the wild felid Leopardus guigna
Source: Vet Res. 2026 Jun 13;57:106. doi: 10.1186/s13567-026-01770-6 (PMC13263956; doi:10.1186/s13567-026-01770-6)

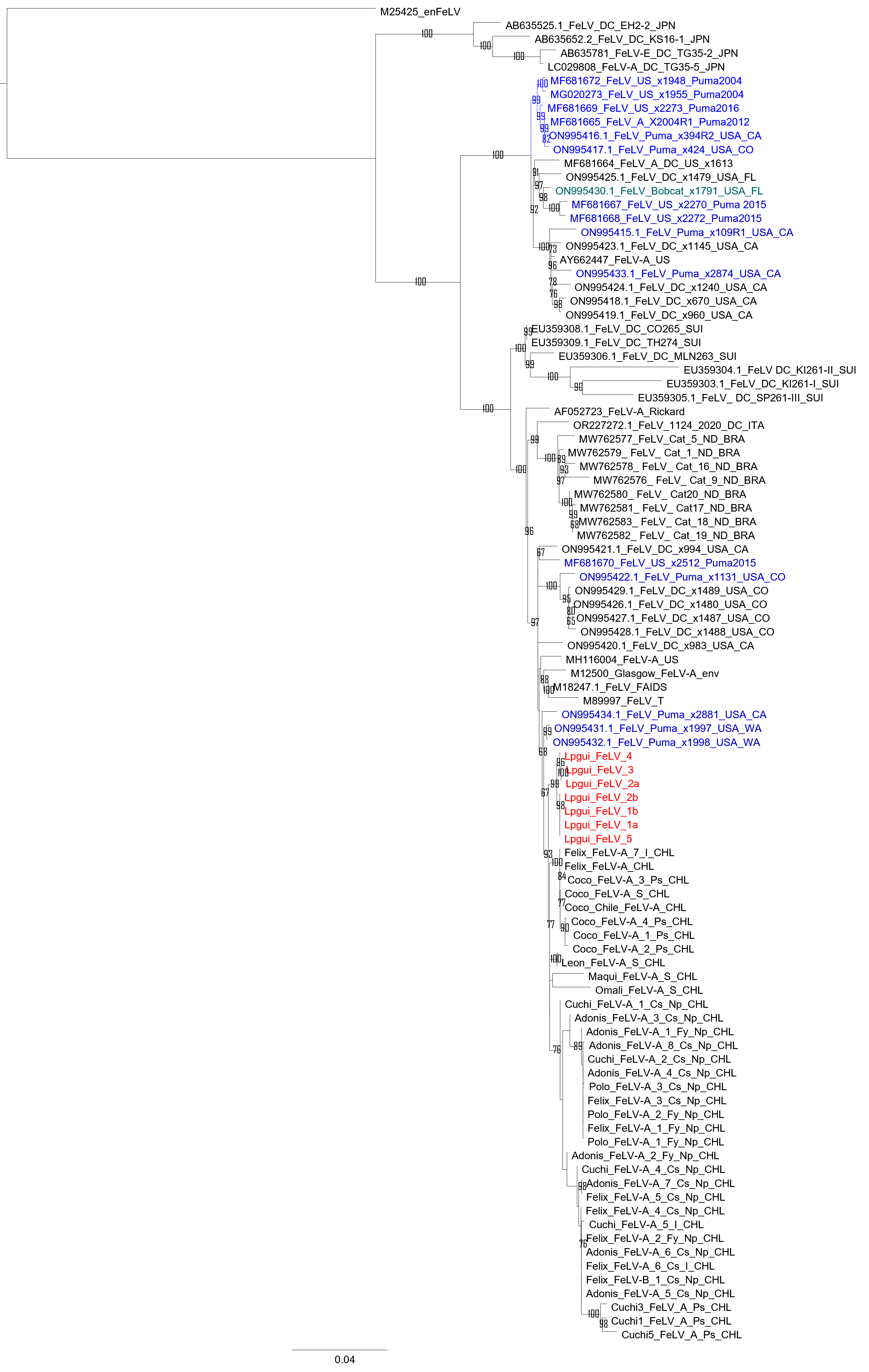

Supplement: Supplementary file 1 — Additional file 1. Uncollapsed phylogenetic tree of the FeLV env genewas constructed using 1000 bootstrap approximations and rooted against enFeLV. The analysis includes only FeLV-A sequences from domestic and nondomestic felids, including all previously reported Chilean sequences. Guigna sequences are shown in red. Puma sequences are shown in blue, and bobcat sequence is shown in turquoise. [file 13567_2026_1770_MOESM1_ESM.png]
